# Supplementary material for: An Experimental Field Trial Investigating the Use of Bacteriophage and Manure Slurry Applications in Beef Cattle Feedlot Pens for Salmonella Mitigation
Source: Animals (Basel). 2023 Oct 11;13(20):3170. doi: 10.3390/ani13203170 (PMC10603643; doi:10.3390/ani13203170)
Supplement: Supplementary file 1 [file animals-13-03170-s001.zip › animals-2562702-supplementary.pdf]

ID#	Tag	WTPen	NewPen	Comments
1	6725	1	N/A	DIED
2	6744	1	N02	WTAMU
3	6747	1	1	USDA/ARS
4	6750	1	1	USDA/ARS
5	6761	1	1	USDA/ARS
6	6762	1	1	USDA/ARS
7	6780	1	1	USDA/ARS
8	6791	1	1	USDA/ARS
9	6824	1	1	USDA/ARS
10	6847	1	1	USDA/ARS
11	6860	1	1	DIED**
12	6861	1	1	USDA/ARS

ID#	Tag	WTPen	NewPen	Comments
1	6715	2	2	USDA/ARS
2	6755	2	N02	WTAMU
3	6764	2	N02	WTAMU
4	6774	2	2	USDA/ARS
5	6798	2	2	USDA/ARS
6	6805	2	2	USDA/ARS
7	6820	2	2	USDA/ARS
8	6825	2	2	USDA/ARS
9	6854	2	2	USDA/ARS
10	6877	2	2	USDA/ARS
11	6896	2	2	USDA/ARS
12	6918	2	2	USDA/ARS

ID#	Tag	WTPen	NewPen	Comments
1	6729	3	N02	WTAMU
2	6735	3	3	USDA/ARS
3	6736	3	3	USDA/ARS
4	6754	3	3	USDA/ARS
5	6799	3	3	USDA/ARS
6	6803	3	3	USDA/ARS
7	6830	3	3	USDA/ARS
8	6832	3	N02	WTAMU
9	6859	3	3	USDA/ARS
10	6900	3	3	USDA/ARS
11	6917	3	3	USDA/ARS
12	6939	3	3	USDA/ARS

ID#	Tag	WTPen	NewPen	Comments
1	6717	4	N/A	DIED
2	6749	4	4	USDA/ARS
3	6765	4	4	USDA/ARS
4	6768	4	4	USDA/ARS
5	6769	4	4	USDA/ARS***
6	6782	4	4	USDA/ARS
7	6806	4	4	USDA/ARS
8	6870	4	4	USDA/ARS
9	6898	4	4	USDA/ARS
10	6908	4	N/A	DIED
11	6916	4	4	USDA/ARS
12	6930	4	4	USDA/ARS

ID#	Tag	WTPen	NewPen	Comments
1	6732	17	5	USDA/ARS
2	6734	17	5	USDA/ARS
3	6738	17	5	USDA/ARS
4	6763	17	5	USDA/ARS
5	6770	17	5	USDA/ARS
6	6771	17	5	USDA/ARS
7	6773	17	5	USDA/ARS
8	6789	17	5	USDA/ARS
9	6795	17	N02	WTAMU
10	6813	17	N02	WTAMU
11	6826	17	5	USDA/ARS
12	6905	17	5	USDA/ARS

ID#	Tag	WTPen	NewPen	Comments
1	6731	18	N02	WTAMU
2	6742	18	6	USDA/ARS
3	6753	18	6	USDA/ARS
4	6760	18	6	USDA/ARS
5	6785	18	6	USDA/ARS
6	6786	18	N02	WTAMU
7	6790	18	6	USDA/ARS
8	6802	18	6	USDA/ARS
9	6808	18	6	USDA/ARS
10	6886	18	6	USDA/ARS
11	6887	18	6	USDA/ARS
12	6911	18	6	USDA/ARS

ID#	Tag	WTPen	NewPen	Comments
1	6719	19	7	USDA/ARS
2	6781	19	7	USDA/ARS
3	6797	19	7	USDA/ARS
4	6842	19	7	USDA/ARS
5	6862	19	7	USDA/ARS
6	6863	19	7	USDA/ARS
7	6867	19	7	USDA/ARS
8	6871	19	N02	WTAMU
9	6873	19	N02	WTAMU
10	6889	19	7	USDA/ARS
11	6902	19	7	USDA/ARS
12	6940	19	7	USDA/ARS

ID#	Tag	WTPen	NewPen	Comments
1	6728	20	8	USDA/ARS
2	6848	20	8	USDA/ARS
3	6882	20	8	USDA/ARS
4	6885	20	8	USDA/ARS
5	6892	20	8	USDA/ARS
6	6919	20	8	USDA/ARS
7	6921	20	N02	WTAMU
8	6924	20	N02	WTAMU
9	6928	20	8	USDA/ARS
10	6931	20	8	USDA/ARS
11	6942	20	8	USDA/ARS
12	6946	20	8	USDA/ARS

ID#	Tag	WTPen	NewPen	Comments
1	6723	48	9	USDA/ARS
2	6737	48	9	USDA/ARS
3	6740	48	9	USDA/ARS
4	6800	48	9	USDA/ARS
5	6818	48	9	USDA/ARS
6	6828	48	9	USDA/ARS
7	6864	48	9	USDA/ARS
8	6866	48	9	USDA/ARS
9	6907	48	9	USDA/ARS
10	6915	48	9	USDA/ARS
11	6925	48	N02	WTAMU
12	6926	48	N02	WTAMU

ID#	Tag	WTPen	NewPen	Comments
1	6720	49	10	USDA/ARS
2	6722	49	N02	WTAMU
3	6727	49	N/A	DIED
4	6741	49	10	USDA/ARS
5	6746	49	10	USDA/ARS
6	6756	49	10	USDA/ARS
7	6775	49	10	USDA/ARS
8	6834	49	10	USDA/ARS
9	6869	49	10	USDA/ARS
10	6879	49	10	USDA/ARS
11	6891	49	10	USDA/ARS
12	6901	49	10	USDA/ARS

ID#	Tag	WTPen	NewPen	Comments
1	6739	50	11	DIED**
2	6743	50	N02	WTAMU
3	6776	50	11	USDA/ARS
4	6784	50	11	USDA/ARS
5	6792	50	11	DIED**
6	6794	50	11	USDA/ARS
7	6844	50	11	USDA/ARS
8	6845	50	11	USDA/ARS
9	6855	50	11	USDA/ARS
10	6865	50	11	USDA/ARS
11	6884	50	11	USDA/ARS
12	6914	50	11	USDA/ARS

ID#	Tag	WTPen	NewPen	Comments
1	6714	51	12	REMOVED
2	6767	51	12	USDA/ARS
3	6810	51	12	USDA/ARS
4	6816	51	12	REMOVED
5	6827	51	12	USDA/ARS
6	6829	51	12	USDA/ARS
7	6858	51	12	USDA/ARS
8	6897	51	12	USDA/ARS***
9	6906	51	12	USDA/ARS
10	6913	51	12	USDA/ARS
11	6923	51	12	USDA/ARS
12	6937	51	12	USDA/ARS

ID#	Tag	WTPen	NewPen	Comments
1	6730	52	13	USDA/ARS
2	6772	52	13	USDA/ARS
3	6793	52	13	USDA/ARS
4	6807	52	13	USDA/ARS
5	6815	52	13	USDA/ARS
6	6823	52	13	USDA/ARS
7	6835	52	13	USDA/ARS
8	6851	52	13	USDA/ARS
9	6872	52	13	USDA/ARS
10	6881	52	N02	WTAMU
11	6904	52	N02	WTAMU
12	6922	52	13	USDA/ARS

ID#	Tag	WTPen	NewPen	Comments
1	6778	53	14	USDA/ARS
2	6783	53	14	USDA/ARS
3	6788	53	14	USDA/ARS
4	6812	53	N02	WTAMU
5	6846	53	N02	WTAMU
6	6850	53	14	USDA/ARS
7	6878	53	14	USDA/ARS
8	6894	53	14	USDA/ARS
9	6899	53	14	USDA/ARS
10	6927	53	14	USDA/ARS
11	6936	53	14	USDA/ARS
12	6938	53	14	USDA/ARS

ID#	Tag	WTPen	NewPen	Comments
1	6718	54	15	USDA/ARS
2	6811	54	15	USDA/ARS
3	6856	54	N02	WTAMU
4	6857	54	15	USDA/ARS
5	6888	54	15	USDA/ARS
6	6890	54	15	USDA/ARS
7	6920	54	15	USDA/ARS
8	6929	54	15	USDA/ARS
9	6935	54	15	USDA/ARS
10	6941	54	15	USDA/ARS
11	6944	54	15	USDA/ARS
12	6945	54	N02	WTAMU

ID#	Tag	WTPen	NewPen	Comments
1	6726	55	16	USDA/ARS
2	6757	55	16	USDA/ARS
3	6796	55	N/A	REMOVED
4	6801	55	16	USDA/ARS
5	6821	55	N02	WTAMU
6	6837	55	16	USDA/ARS
7	6840	55	16	USDA/ARS
8	6868	55	16	USDA/ARS
9	6876	55	16	USDA/ARS
10	6880	55	16	USDA/ARS
11	6910	55	16	USDA/ARS
12	6943	55	16	USDA/ARS

ID#	Tag	WTPen	NewPen	Comments
1	6716	56	17	USDA/ARS
2	6733	56	N02	WTAMU
3	6751	56	17	USDA/ARS
4	6758	56	17	USDA/ARS
5	6822	56	17	USDA/ARS
6	6833	56	N02	WTAMU
7	6836	56	17	USDA/ARS
8	6838	56	17	USDA/ARS
9	6849	56	17	USDA/ARS
10	6874	56	17	USDA/ARS
11	6893	56	N/A	DIED
12	6909	56	17	USDA/ARS
N/A	6912	46	17	USDA/ARS*

ID#	Tag	WTPen	NewPen	Comments
1	6721	57	18	USDA/ARS
2	6779	57	18	USDA/ARS
3	6814	57	18	USDA/ARS
4	6817	57	N02	WTAMU
5	6819	57	18	USDA/ARS
6	6831	57	18	USDA/ARS
7	6853	57	18	USDA/ARS
8	6903	57	N02	WTAMU
9	6932	57	18	USDA/ARS
10	6933	57	18	USDA/ARS
11	6934	57	18	USDA/ARS
12	6947	57	18	USDA/ARS

Figure S1. Trial cattle (Tag) pen placements at WTAMU Feedlot (WTPen) and AgriLife Feedlot (NewPen). The comments section indicates if cattle remained at the WTAMU Feedlot, were part of the trial at the AgriLife Feedlot, died or, were removed from the study due to illness. *An extra animal was brought in to replace one that died.

Table S1. Isolates selected for the *Salmonella* cocktails for natural-phage enrichment from the pen environment.

Isolate ID	Sample Origin	Serovar	NCBI Accession Number
Cocktail #1 - Levent et al., (2019) Isolates [22]			
3-day99-58-4347-10-ISO1	Hide	Lubbock	SAMN10910316
3-day99-58-4268-11-ISO1	Hide	Cerro	SAMN10910317
3-day120-54-4263-ISO2	Hide	Anatum	SAMN10910329
3-day-day134-9-4220-10-ISO1	Hide	Montevideo	SAMN10910378
1-day141-53-4331-5-ISO1	Fecal Grab	Newport	SAMN10910195
1-day7-9-4243-5-ISO1	Fecal Grab	Kentucky	SAMN10910075
Cocktail #2 - Experimental Trial Isolates			
2-D7-P7-1	Pen Environment	Muenster	SAMN31536193
2-D28-P3-1	Pen Environment	Newport	SAMN31536677
7-D14-P2-6825	Brisket Swab	61:l,v:1,5	SAMN31536359
1-D21-P8-6919	Brisket Swab	Kentucky	SAMN31536373
7-D14-P7-6867	Brisket Swab	Lille	SAMN31536429
8-D21-P6-6785	Rump Swab	Virginia	SAMN31536567

Table S2. *Salmonella* experimental trial isolates used as bacterial lawns for spot testing of Pen 52 to Pen 55 virome.

Isolate ID	Sample Origin	Serovar	NCBI Accession Number
2-D7-P7-1	Pen Environment	Muenster	SAMN31536193
2-D28-P3-1	Pen Environment	Newport	SAMN31536677
1-D14-P1-6750	Fecal Grab	Montevideo	SAMN31536297
7-D14-P2-6825	Brisket Swab	61:l,v:1,5	SAMN31536359
7-D7-P3-6900	Brisket Swab	Anatum	SAMN31536196
1-D14-P4-6870	Fecal Grab	Cerro	SAMN31536308
8-D21-P6-6785	Rump Swab	Virginia	SAMN31536567
7-D14-P7-6867	Brisket Swab	Lille	SAMN31536373
1-D21-P8-6919	Fecal Grab	Kentucky	SAMN31536429
8-D7-P12-6810	Rump Swab	Lubbock	SAMN31536276

Table S3. WTAMU Feedlot longitudinal study *Salmonella* isolates (n=31) used in bacteriophage host range testing. Isolates are from Nickodem et al., (2023) [20]

Isolate ID	Sample Origin	Serovar	NCBI Accession Number
4-jul-P11-1	Water	Senftenberg	SAMN25996410
4-jul-P9-1	Water	Derby	SAMN25996404
2-jun-P59-1	Pen Environment	Virginia	SAMN25996101
6-deca-sq109-1	Lymph Node	Virginia	SAMN25996518
6-deca-sq147-1	Lymph Node	Virginia	SAMN25996523
6-deca-sq86-1	Lymph Node	Virginia	SAMN25996529
1-jun-P21-3	Freshly Voided Feces	Cerro	SAMN25996078
5-aug-P12-1	Feed	Cerro	SAMN25996562
4-oct-P11-1	Water	Cerro	SAMN25996512
2-dec-P22-1	Pen Environment	Cerro	SAMN25996327
6-deca-sq101-1	Lymph Node	Cerro	SAMN25996516
1-jul-P50-4	Freshly Voided Feces	Lubbock	SAMN25996155
2-aug-P34-1	Pen Environment	Lubbock	SAMN25996167

2-sept-P42-1	Pen Environment	Lubbock	SAMN25996396
1-oct-P29-3	Freshly Voided Feces	Lubbock	SAMN25996377
6-deca-sq128-1	Lymph Node	Lubbock	SAMN25996452
5-aug-P2-1	Feed	Anatum	SAMN25996428
1-sept-P10-4	Freshly Voided Feces	Anatum	SAMN25996195
4-oct-P20-1	Water	Anatum	SAMN25996513
6-oct-sq16-1	Lymph Node	Anatum	SAMN25996443
2-nov-P19-1	Pen Environment	Anatum	SAMN25996276
4-jun-P58-1	Water	Kentucky	SAMN25996557
2-sept-P32-1	Pen Environment	Kentucky	SAMN25996235
6-oct-sq7-1	Lymph Node	Kentucky	SAMN25996446
1-nov-P1-1	Freshly Voided Feces	Kentucky	SAMN25996342
1-nov-P23-4	Freshly Voided Feces	Kentucky	SAMN25996350
2-jun-P4-1	Pen Environment	Montevideo	SAMN25996097
5-jul-P30-1	Feed	Montevideo	SAMN25996430
4-aug-P28-1	Water	Montevideo	SAMN25996556
1-sept-P41-3	Freshly Voided Feces	Montevideo	SAMN25996214
2-nov-P33-1	Pen Environment	Montevideo	SAMN25996553
2-D7-P7-1	Pen Environment	Muenster	SAMN31536193
2-D28-P8-1	Pen Environment	Muenster	SAMN31536687
1-D14-P8-6931	Fecal Grab	Muenster	SAMN31536317
8-D21-P9-6737	Rump Swab	Muenster	SAMN31536577
7-D21-P10-6756	Brisket Swab	Muenster	SAMN31536490
1-D14-P1-6861	Fecal Grab	Newport	SAMN31536298
2-D28-P3-1	Pen Environment	Newport	SAMN31536677
2-D14-P9-1	Pen Environment	Newport	SAMN31536356
7-D21-P9-6740	Brisket Swab	Newport	SAMN31536516
8-D21-P10-6869	Rump Swab	Newport	SAMN31536555

Table S4. The overall *Salmonella* prevalence of all cattle (n = 216) and feedlot pen environment (n = 18) included in the pre-trial period by sample type and day.

Day	Fecal	Brisket Swabs	Rump Swabs	Pen Environment	Pen Environment PNO2**
Day -43	N/A	N/A	N/A	27.8% (5/18)	N/A
Day -36	19.9% (43/216)	77.3% (167/216)	81.9% (177/216)	38.9% (7/18)	N/A
Day -21	11.2% (24/215)	45.6% (98/215)	23.7% (51/215)	66.7% (12/18)	N/A
Day -7*	36.7% (77/210)	80.3% (167/208)	81.7% (170/208)	77.8% (14/18)	100% (3/3)

*On Day -7, one cattle died after initial sampling and a new one from extra cattle was sampled and added
**PNO2 was sampled on Day -7 prior to cattle relocation (n=28) to this pen for follow-up sampling

Table S5. Pre-trial *Salmonella* prevalence of the subset of cattle (n=180) included in the trial by sample type, day, and future treatment group.

Day	Sample	Manure Slurry Group	Phage Cocktail Group	Combination Group*	Control Group**
Day -36	Feces	27.5% (11/40)	12.5% (5/40)	24.5% (12/49)	18.4% (9/49)
	Brisket	65.0% (26/40)	90.0% (36/40)	75.5% (27/49)	75.5% (37/49)
	Rump	77.5% (31/40)	77.5% (31/40)	83.7% (41/49)	85.7% (42/49)
	Avg.	56.7% (68/120)	60.0% (72/120)	54.4% (80/147)	59.9% (88/147)
Day -21	Feces	10.0% (4/40)	12.5% (5/40)	14.3% (7/49)	12.2% (6/49)
	Brisket	45.0% (18/40)	35.0% (14/40)	59.2% (29/49)	36.7% (18/49)
	Rump	30.0% (12/40)	12.5% (5/40)	36.7% (18/49)	14.3% (7/49)
	Avg.	28.3% (34/120)	20.0% (24/120)	36.7% (54/147)	21.1% (31/147)
Day -7	Feces	35.0% (14/40)	15.0% (6/40)	42.9% (21/49)	55.1% (27/49)
	Brisket	72.5% (29/40)	60.0% (24/40)	95.9% (47/49)	81.6% (40/49)
	Rump	75.0% (30/40)	77.5% (31/40)	89.8% (44/49)	79.6% (39/49)
	Avg.	60.8% (73/120)	50.8% (61/120)	76.2% (112/147)	72.1% (106/147)

*There were only 9 cattle in Pen 1. **There were only 9 cattle in Pen 11

Table S6. Experimental trial cattle *Salmonella* prevalence (n=180) by day, sample type, and treatment group.

Day	Sample	Manure Slurry Group	Phage Cocktail Group	Combination Group	Control Group
Day -7 (Incoming Prevalence)	Feces	35.0% (14/40)	15.0% (6/40)	42.9% (21/49)	55.1% (27/49)
	Brisket	72.5% (29/40)	60.0% (24/40)	95.9% (47/49)	81.6% (40/49)
	Rump	75.0% (30/40)	77.5% (31/40)	89.8% (44/49)	79.6% (39/49)
Day 0 (Baseline Prevalence)	Feces**	0.0% (0/33)	2.6% (1/38)	17.4% (8/46)	22.9% (11/48)
	Brisket	22.5% (9/40)	35.0% (14/40)	38.8% (19/49)	69.4% (34/49)
	Rump	15.0% (6/40)	20.0% (8/40)	22.4% (11/49)	30.6% (15/49)
Day 7*	Feces	15.0% (6/40)	7.5% (3/40)	22.5% (11/49)	30.6% (15/49)
	Brisket	45.0% (18/40)	30.0% (12/40)	30.6% (15/49)	61.2% (30/49)
	Rump	17.5% (7/40)	0.0% (0/40)	18.4% (9/49)	10.2% (5/49)
Day 14*	Feces	17.5% (7/40)	10.0% (4/40)	16.3% (8/49)	20.4% (10/49)
	Brisket	25.0% (10/40)	47.5% (19/40)	20.4% (10/49)	51.0% (25/49)
	Rump	37.5% (15/40)	47.5% (19/40)	26.5% (13/49)	73.5% (36/49)
Day 21*	Feces	10.0% (4/40)	20.0% (8/40)	10.2% (5/49)	32.7% (16/49)
	Brisket	5.0% (2/40)	7.5% (3/40)	4.1% (2/49)	26.5% (13/49)
	Rump	0.0% (0/40)	5.0% (2/40)	2.0% (1/49)	34.7% (17/49)
Days 7-21 Avg. (Post-Treatment Prevalence)	Feces	14.2% (17/120)	12.5% (15/120)	16.3% (24/147)	27.9% (41/147)
	Brisket	25.0% (30/120)	28.3% (34/120)	18.4% (27/147)	46.3% (68/147)
	Rump	18.3% (22/120)	17.5% (21/120)	15.6% (23/147)	39.5% (58/147)

*Treatment applications started Day 1 of the trial; therefore, only Days 7-Day 21 can be considered for the impact of treatment. **There were 13 missing fecal grab samples across Day 0 treatment groups

Table S7. Multilevel mixed effects logistic regression model outputs by sample type.

Sample Type	Model	−2 (log-likelihood)	*p*-value
Fecal Grab	Intercept	903.675	.
	Feedlot pen	836.890	0.0000
	Manure slurry, phage cocktail, day	819.686	0.0000
	Manure slurry, phage cocktail, day, envi	815.529	0.0000
	3-way Interaction (manure slurry, phage cocktail, day)	763.010	0.0000
	3-way full-factorial (manure slurry, phage cocktail, day), pen environment	746.606	0.0002
	3-way full-factorial (manure slurry, phage cocktail, day), 2-way interaction (pen environment, day)	743.570	0.0002
	3-way Interaction (manure slurry phage cocktail, day), tag as random effect	746.414	0.0000
	3-way full-factorial (manure slurry, phage cocktail, day), pen environment, tag as random effect	729.850	0.0000
	3-way full-factorial (manure slurry, phage cocktail, day), 2-way interaction (pen environment, day), tag as random effect	726.771	0.0000
Brisket Swab	Intercept	1,211.688	
	Feedlot Pen	1,148.139	0.0000
	Manure slurry, phage cocktail, day	968.104	0.0000
	Manure slurry, phage cocktail, day, envi	967.690	0.0000
	3-way Interaction (manure slurry, phage cocktail, day)	882.758	0.0000
	3-way full-factorial (manure slurry phage cocktail, day), pen environment	882.758	0.0000
	3-way full-factorial (manure slurry, phage cocktail, day), 2-way interaction (pen environment, day)	866.932	0.0000
	3-way Interaction (manure slurry, phage cocktail, day), tag as random effect	882.758	0.0000
	3-way full-factorial (manure slurry, phage cocktail, day), pen environment, tag as random effect	882.758	0.0000
	3-way full-factorial (manure slurry, phage cocktail, day), 2-way interaction (pen environment, day), tag as random effect	866.932	0.0000
Rump Swab	Intercept	1,148.061	
	Feedlot Pen	1,091.080	0.0000
	Manure slurry phage cocktail, day	966.777	0.0000
	Manure slurry, phage cocktail, day, envi	962.323	0.0000
	3-way Interaction (manure slurry, phage cocktail, day)	694.867	0.0000
	3-way full-factorial (manure slurry, phage cocktail, day), pen environment	691.538	0.0000
	3-way full-factorial (manure slurry, phage cocktail, day), 2-way interaction (pen environment, day)	688.653	0.0000
	3-way Interaction (manure slurry, phage cocktail, day), tag as random effect	694.867	0.0000

	3-way full-factorial (manure slurry, phage cocktail, day), pen environment, tag as random effect	691.537	0.0000
	3-way full-factorial (manure slurry, phage cocktail, day), 2-way interaction (pen environment, day), tag as random effect	688.652	0.0000

The AgriLife Feedlot pen was included as a random effect for all models except for the intercept-only model. For fecal grab, brisket swab, and rump swab the final model included *Salmonella* prevalence as the dependent variable, feedlot pen as the random effects variable, and the 3-way full-factorial (manure slurry, phage cocktail, collection day) term and 2-way interaction term (pen environment (indicating environment *Salmonella* status) and day) were included as fixed effects.

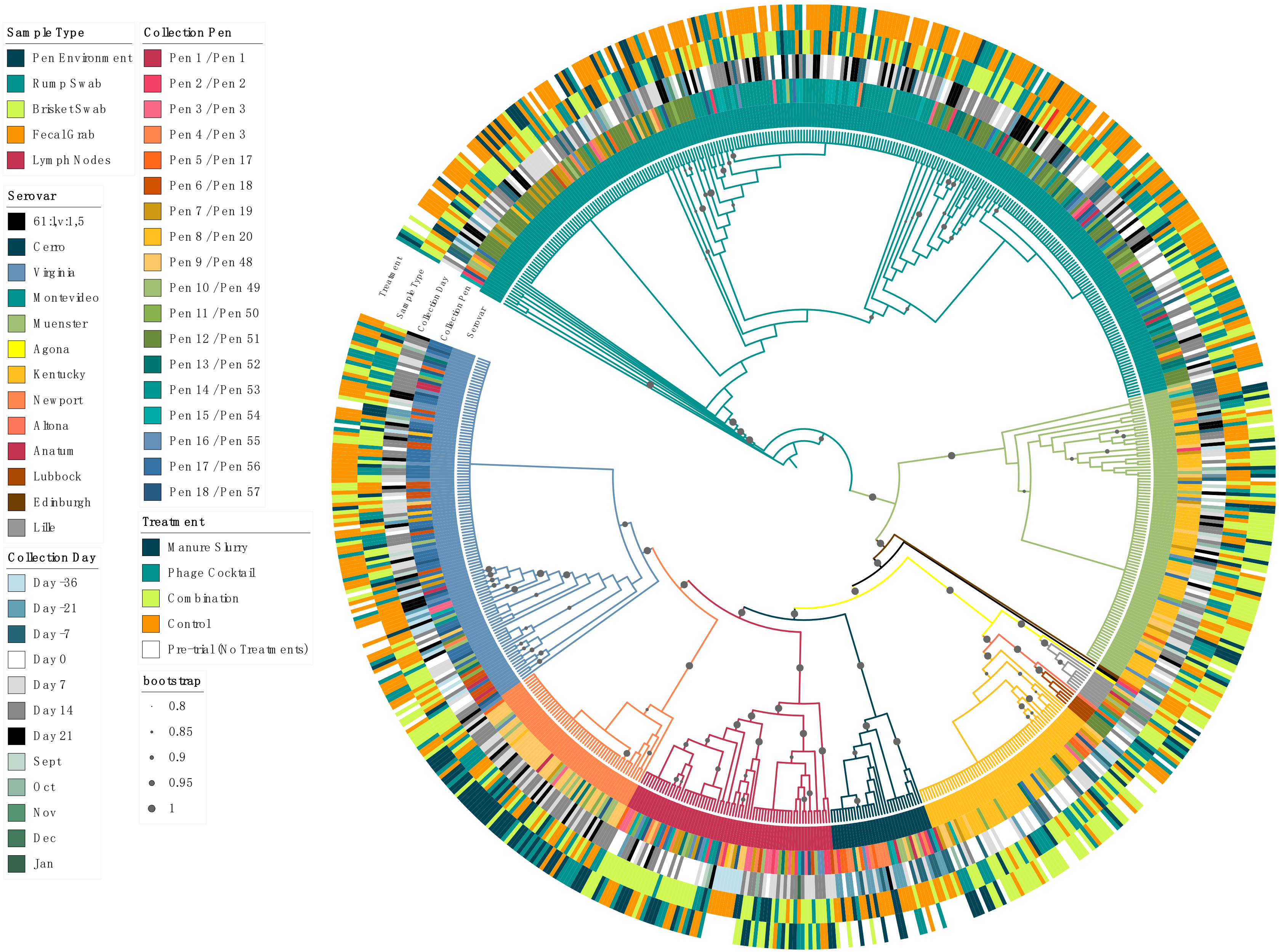
Sample Type
Pen Environment
Rump Swab
Brisket Swab
Fecal Grab
Lymph Nodes
Serovar
61:l,v:1,5
Cerro
Virginia
Montevideo
Muenster
Agona
Kentucky
Newport
Altona
Anatum
Lubbock
Edinburgh
Lille
Collection Day
Day -36
Day -21
Day -7
Day 0
Day 7
Day 14
Day 21
Sept
Oct
Nov
Dec
Jan
Collection Pen
Pen 1 / Pen 1
Pen 2 / Pen 2
Pen 3 / Pen 3
Pen 4 / Pen 3
Pen 5 / Pen 17
Pen 6 / Pen 18
Pen 7 / Pen 19
Pen 8 / Pen 20
Pen 9 / Pen 48
Pen 10 / Pen 49
Pen 11 / Pen 50
Pen 12 / Pen 51
Pen 13 / Pen 52
Pen 14 / Pen 53
Pen 15 / Pen 54
Pen 16 / Pen 55
Pen 17 / Pen 56
Pen 18 / Pen 57
Treatment
Manure Slurry
Phage Cocktail
Combination
Control
Pre-trial (No Treatments)
bootstrap
0.8
0.85
0.9
0.95
1
Treatment
Sample Type
Collection Day
Collection Pen
Serovar

Figure S2. Phylogenetic tree of all *Salmonella* isolates (n = 717) across the pre-trial (n = 117), trial (n = 557), and follow-up (n = 43) sampling periods. Collection pen represents the cattle that were grouped together in pens at the AgriLife Feedlot (left in the legend) and WTAMU Feedlot (right in the legend), which are shaded by block. Manure slurry (navy blue), phage cocktail (teal), combination (bright green), control (orange), and isolates from the pre-trial period before treatments began (white) are represented in the third ring. The pre-trial (Days -36 through -7; blue shades) occurred at the WTAMU Feedlot, the trial (Days 0 through 21; grayscale) occurred at the AgriLife Feedlot, and only the follow-up isolates (September through January; green shades) from the AgriLife Feedlot are included. Sample type is represented in the outermost ring. Bootstrap values ranging from 0.8 to 1 were included in the tree.
